# Branched-chain amino acid aminotransferase and methionine formation in *Mycobacterium tuberculosis*

**DOI:** 10.1186/1471-2180-4-39

**Published:** 2004-10-07

**Authors:** Erik S Venos, Marvin H Knodel, Cynthia L Radford, Bradley J Berger

**Affiliations:** 1 Chemical & Biological Defence Section, Defence R&D Canada – Suffield, Box 4000 Station Main, Medicine Hat, Alberta, T1A 8K6, Canada

## Abstract

**Background:**

Tuberculosis remains a major world-wide health threat which demands the discovery and characterisation of new drug targets in order to develop future antimycobacterials. The regeneration of methionine consumed during polyamine biosynthesis is an important pathway present in many microorganisms. The final step of this pathway, the conversion of ketomethiobutyrate to methionine, can be performed by aspartate, tyrosine, or branched-chain amino acid aminotransferases depending on the particular species examined.

**Results:**

The gene encoding for branched-chain amino acid aminotransferase in *Mycobacterium tuberculosis *H37Rv has been cloned, expressed, and characterised. The enzyme was found to be a member of the aminotransferase IIIa subfamily, and closely related to the corresponding aminotransferase in *Bacillus subtilis*, but not to that found in *B. anthracis *or *B. cereus*. The amino donor preference for the formation of methionine from ketomethiobutyrate was for isoleucine, leucine, valine, glutamate, and phenylalanine. The enzyme catalysed branched-chain amino acid and ketomethiobutyrate transamination with a Km of 1.77 – 7.44 mM and a Vmax of 2.17 – 5.70 μmol/min/mg protein, and transamination of ketoglutarate with a Km of 5.79 – 6.95 mM and a Vmax of 11.82 – 14.35 μmol/min/mg protein. Aminooxy compounds were examined as potential enzyme inhibitors, with O-benzylhydroxylamine, O-t-butylhydroxylamine, carboxymethoxylamine, and O-allylhydroxylamine yielding mixed-type inhibition with Ki values of 8.20 – 21.61 μM. These same compounds were examined as antimycobacterial agents against *M. tuberculosis *and a lower biohazard *M. marinum *model system, and were found to completely prevent cell growth. O-Allylhydroxylamine was the most effective growth inhibitor with an MIC of 78 μM against *M. marinum *and one of 156 μM against *M. tuberculosis*.

**Conclusion:**

Methionine formation from ketomethiobutyrate is catalysed by a branched-chain amino acid aminotransferase in *M. tuberculosis*. This enzyme can be inhibited by selected aminooxy compounds, which also have effectiveness in preventing cell growth in culture. These compounds represent a starting point for the synthesis of branched-chain aminotransferase inhibitors with higher activity and lower toxicity.

## Background

Tuberculosis remains one of the leading causes of worldwide mortality and morbidity, infecting an estimated 8 million people annually with approximately 2 million deaths [1]. The situation regarding the control of tuberculosis has significantly worsened over the last decades, with the spread of multidrug resistant strains. In the absence of an effective vaccine for tuberculosis, there is an urgent need for the development of novel antimycobacterial agents. The study of mycobacterial biochemistry assists this development through the identification and characterization of cellular enzymes amenable to therapeutic inhibition.

**Figure 1:**
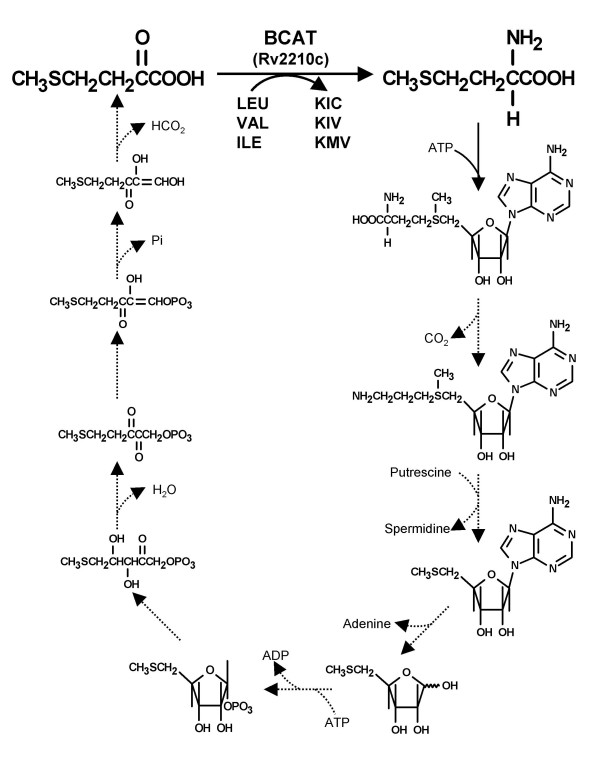
**The formation of Met from KMTB. **The pathway of polyamine synthesis and subsequent Met regeneration from MTA, as known from *K. pneumoniae *[11] and *B. subtilis *[12], is shown. Solid arrows represent steps that have been characterised in *M. tuberculosis *(present study and [21]). The conversion of KMTB to Met is shown at the top in bold. KIC = ketoisocaproate, KIV = ketoisovalerate, and KMV = ketomethylvalerate.

Polyamine synthesis and its associated methionine (Met) regeneration pathway (Figure 1) are known to be potential drug targets in a variety of microorganisms [2-4]. The synthesis of polyamines is essential during periods of DNA replication, although the exact physiological role of these compounds remains unclear [3]. The production of spermidine from putrescine, or spermine from spermidine, consumes the amino acid Met in a 1:1 stoichiometry yielding methylthioadenosine (MTA) as a byproduct. As Met biosynthesis is energetically expensive, and many organisms lack the ability to synthesize the amino acid, a unique pathway exists which recycles Met from MTA. To date, the entire pathway has only been fully characterised in the Gram-negative bacterium *Klebsiella pneumoniae *[5-11] and the Gram-positive bacterium *Bacillus subtilis *[12-14] Selected individual enzymes active in the pathway have been studied in a wide variety of eukaryotic and prokaryotic organisms [7,15-20]. For *Mycobacterium spp*., only methionine adenosyltransferase has been cloned, expressed, and fully characterised [21].

The final step in Met regeneration is the transamination of ketomethiobutyrate (KMTB) by an aminotransferase. The specific aminotransferase responsible for the reaction has been identified and characterised in a number of microorganisms, including malaria, African trypanosomes, *K. pneumoniae*, *B. subtilis*, and *B. anthracis *[7,16,17]. In the lower eukaryotes *Plasmodium falciparum*, *Trypanosoma brucei brucei*, *Giardia intestinalis*, and *Crithidia fasciculata*, this reaction is catalysed by the subfamily Ia enzyme aspartate aminotransferase [17]. In *K. pneumoniae*, however, the reaction was performed by the close homologue tyrosine aminotransferase, which is also a member of subfamily Ia [7]. Gram-positive bacteria and archaea appear to lack any subfamily Ia homologues in their genomes, and *B. subtilis*, *B. cereus*, and *B. anthracis *were recently found to catalyse Met regeneration via a branched-chain amino acid aminotransferase (BCAT) [16]. This enzyme is a member of family III, along with D-amino acid aminotransferase (DAAT), and is unrelated structurally to family I enzymes [22]. Intriguingly, *B. subtilis *and *B. cereus*/*B. anthracis *utilised BCAT enzymes from separate subfamilies (IIIa vs. IIIb respectively). As *Mycobacterium spp. *also appear to have no subfamily Ia aminotransferase sequences ([16], and data not shown), it would be expected that *M. tuberculosis *also catalyses the conversion of KMTB to Met via a BCAT. In this paper, we report the identification, cloning, and functional expression of a single BCAT from *M. tuberculosis*. In addition, this enzyme has been demonstrated to actively catalyse Met formation and is subject to inhibition by a variety of aminooxy compounds.

## Results

### Branched-chain amino acid aminotransferase in *M. tuberculosis*

**Figure 2:**
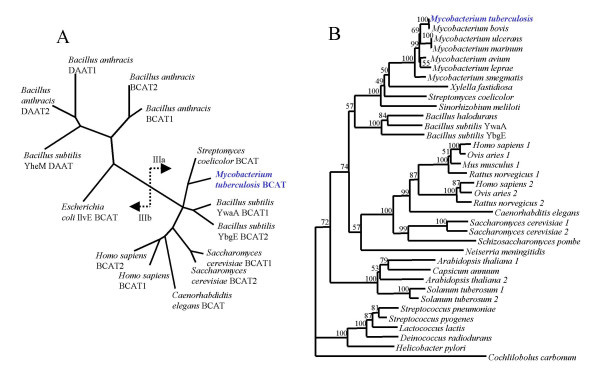
**Relationship of *M. tuberculosis *BCAT to other family III aminotransferases. **In (A) selected subfamily IIIa and IIIb aminotransferases were aligned and a tree constructed by the neighbor-joining method [49] in order to define the subfamily membership of the *M. tuberculosis *BCAT (in blue). In (B) subfamily IIIa BCATs were aligned and a cladogram constructed by the neighbor-joining method. The numbers represent the bootstrap values (in percentage) for each branch point.

The complete, published genome of *M. tuberculosis *H37Rv was found to contain a single gene with a very high sequence homology to either *B. subtilis *YbgE or YwaA, which are both known to be subfamily IIIa BCATs [16,23]. In contrast, the tuberculosis genome did not contain a homologue to *B. subtilis *YheM, *B. cereus *BCAT, or *B. anthracis *BCAT, which are all subfamily IIIb aminotransferases [16]. This relationship can be clearly seen in Figure 2A, where selected family III aminotransferases have been aligned and an unrooted tree constructed. The putative *M. tuberculosis *BCAT gene, Rv2210c, has not been previously cloned, expressed, or characterised. It is interesting to note that the *M. tuberculosis *genome contains a single BCAT homologue and no obvious DAAT homologue.

Examination of complete and incomplete genome projects for *Mycobacterium spp*. uncovered a single gene in *M. leprae*, *M. bovis*, *M. marinum*, *M. ulcerans*, *M. avium*, and *M. smegmatis *with an extremely high identity to Rv2110c. Together, with other subfamily IIIa aminotransferases, the putative mycobacterial sequences were aligned and a cladogram constructed (Figure 2B). The *M. tuberculosis *and *M. bovis *sequences were identical, as were the *M. marinum *and *M. ulcerans *sequences. Aside from *M. bovis*, all the mycobacterial BCAT sequences were found to be 85 – 88% identical to the *M. tuberculosis *sequence. However, the tuberculosis sequence was 57% identical to the putative BCAT from *Streptomyces coelicolor *and 45% identical to *B. subtilis *YbgE. Figure 2B highlights the fact that the mycobacterial BCAT sequences are more closely related to eukaryotic enzymes than to most other bacterial homologues. There was little sequence conservation with enzymes found in subfamily IIIb, with only 27% identity to the *E. coli *BCAT, 18% to the *B. anthracis *BCAT, and 15% to *B. subtilis *YheM.

**Figure 3:**
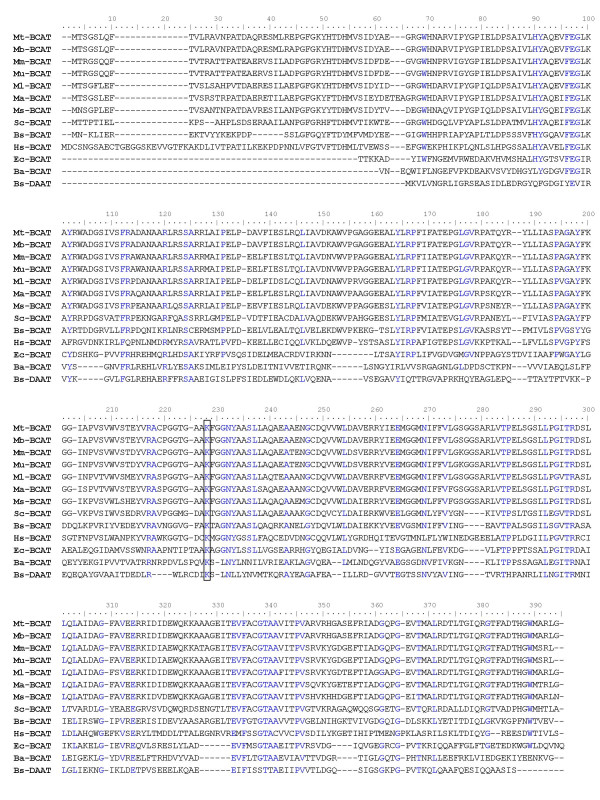
**Alignment of selected family III aminotransferases. **The following sequences were aligned with the Clustal algorithm: Mt-BCAT, *M. tuberculosis *BCAT [23]; Mb-BCAT, *M. bovis *BCAT [51]; Mm-BCAT, *M. marinum *BCAT; Mu-BCAT, *M. ulcerans *BCAT; Ml-BCAT, *M. leprae *BCAT [52]; Ma-BCAT, *M. avium *BCAT; Ms- BCAT, *M. smegmatis *BCAT; Sc- BCAT, *Streptomyces coelicolor *BCAT [53]; Bs-BCAT, *Bacillus subtilis *BCAT YbgE [43]; Hs-BCAT1, human BCAT1 [54]; Ec-BCAT, *Escherichia coli *BCAT IlvE [55]; Ba-BCAT, *B. anthracis *BCAT2 [16]; Bs-DAAT, *B. subtilis *DAAT YheM [43]). Residues conserved by 80% of the sequences are shown in blue. The boxed residues represent the pyridoxal-5-phosphate binding site.

The low level of sequence conservation outside of the genus can be seen in the alignment of selected BCAT sequences shown in Figure 3. Only 19 residues are completely conserved across even this small sequence sampling. Interestingly, of the residues found by X-ray crystallography to be important in substrate binding to the *E. coli *BCAT [24], only K228(K159) and T339(T257) were conserved across the 13 sequences in Figure 3. The residues in parentheses represent the corresponding position in the *E. coli *BCAT. Of these two residues, K228(K159) is the PLP binding site and would be expected to be invariant. If one excludes the only DAAT in Figure 3, then Y91(Y31), F96(36), Y233(164), and A340(A258) can be added to this conserved list of residues important for substrate binding in the *E. coli *BCAT. Clearly, sequence conservation is very low across family III.

### Expression and characterization of the branched-chain amino acid aminotransferase

**Figure 4:**
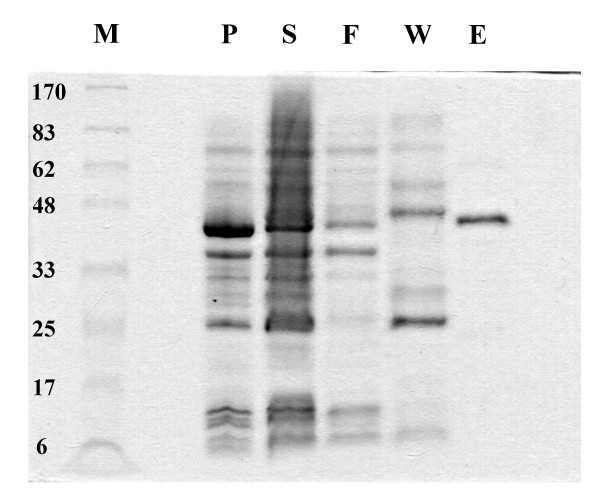
**Purification of recombinant *M. tuberculosis *BCAT. ***E. coli *BL21(DE3) CodonPlus-RIL cells were induced with IPTG and prepared as described in the Materials and Methods section. The cell lysate was separated by centrifugation into pellet (P) and supernatant (S) fractions. The supernatant was loaded onto an Ni^2+^-charged metal ion affinity column and flow through (F), 80 mM imidazole (W), and 800 mM imidazole (E) fractions were collected. Aliquots of each fraction were analysed on a 10% polyacrylamide gel under reducing conditions. Lane (M) contains molecular mass markers (units in kDa).

The putative *M. tuberculosis *BCAT was cloned as a deca-histidine fusion protein for expression in *E. coli*. To prevent complete inclusion of the recombinant protein, it was necessary to induce expression with a relatively low concentration of IPTG (0.1 mM) at 20°C for 20 hr. Under these conditions, sufficient soluble material was produced and purified over Ni^2+ ^affinity columns (Figure 4). Assay of the eluted material with 2 mM each of ADEFGHIKLNQRSTVWY and 1 mM KMTB resulted in appreciable Met production (data not shown), demonstrating that the enzyme was active and catalysed Met formation.

**Figure 5:**
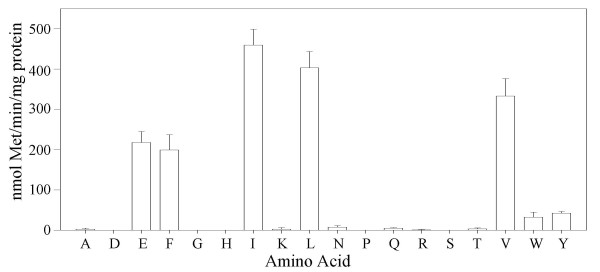
**The amino donor range for Met formation. **The enzyme was mixed with 1.0 mM KMTB, 2.0 mM of an individual amino acid, and PLP for 30 min at 37°C before HPLC analysis of Met production.

The purified enzyme was screened against 2 mM of each individual amino acid and 1 mM KMTB to determine the amino donor range for Met regeneration. Isoleucine, leucine, and valine were found to be the most effective substrates (Figure 5), while glutamate and phenylalanine were also active as amino donors. Tyrosine and tryptophan were found to have a much lesser ability to transaminate KMTB and all other amino acids were inactive. The five most active amino donors were more closely examined in order to determine their kinetic parameters (Table 1). The Km for Leu, Ile, and Val ranged from 1.77 – 2.85 mM, while that for Glu was 9.53 mM and Phe 7.44 mM. The Vmax for all five amino acids was similar at 2.17 – 5.70 μmol/min/mg protein. KMTB was found to have a Km of 4.20 mM. The enzyme was also examined for branched-chain amino acid and KG aminotransfer in order characterise the "classic" reactions associated with a BCAT (Table 1). The Km of the substrates was found to be similar, while the Vmax ranged from 11.82 – 14.35 μmol/min/mg protein. Therefore, the tuberculosis BCAT catalyses aminotransfer of KG about 3 times more readily than KMTB. This result is similar to that seen with the *B. subtilis *BCAT, which also transaminates KG at a higher rate than KMTB [16].

### Inhibition studies

**Figure 6:**
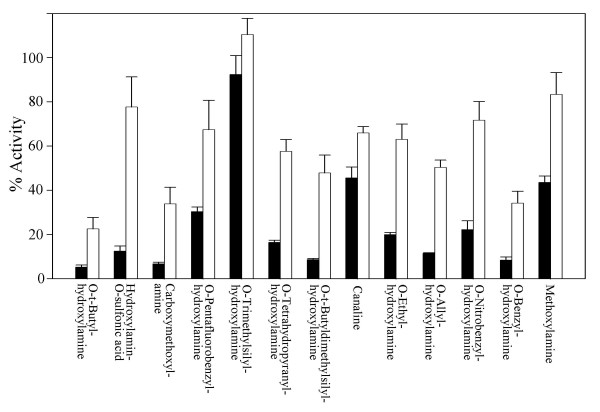
**Inhibition of branched-chain aminotransferase by aminooxy compounds. **Leucine, KMTB, PLP and 1 mM (black bars) or 0.1 mM (white bars) of inhibitor were incubated with Mt-BCAT as described in the Methods section. Percent activity is shown relative to a positive control which contained no inhibitor.

Thirteen aminooxy compounds were assayed for inhibitory effects on the tuberculosis BCAT. The enzyme was incubated with 2.0 mM leucine, 1.0 mM KMTB and 0.1 or 1.0 mM inhibitor to assay for the effect on Met regeneration (Figure 6). With the exception of O-trimethylsilylhydroxylamine, all of the compounds inhibited Met formation to some extent. The four most active compounds at 0.1 mM were O-allylhydroxylamine, carboxymethoxylamine, O-benzylhydroxylamine, and O-t-butylhydroxylamine, and these inhibitors were further examined in order to determine K_i _values (Table 2). For all four compounds, the inhibition data was not consistent with a simple competitive or uncompetitive model, but fit very well with a model of mixed mode inhibition [25]. The competitive component of inhibition yielded a K_ic _of 8.20 – 21.61 μM, while the uncompetitive component gave a K_iu _of 84.08 – 386 μM. Therefore, the inhibition of the tuberculosis BCAT by these four aminooxy compounds is primarily competitive.

These four inhibitors and canaline, an aminooxy analogue of ornithine that has been demonstrated to be an effective aminotransferase inhibitor in other systems [16,17,26-28], were screened against *M. tuberculosis *and *M. marinum *in vitro to determine potential antimicrobial activity. *M. marinum *is a close relative of *M. tuberculosis *that causes a similar disease in fish, grows faster than *M. tuberculosis *in culture, and does not cause serious infections in humans [29]. As such, it is an excellent surrogate for the initial screening of antimycobacterial agents, and we wished to validate its use for aminooxy compounds. All the inhibitors were found to have some degree of antimycobacterial activity (Table 3), with MIC values ranging from 78 μM – 10 mM and IC_50 _values of 8.49 μM – 467 μM. The best inhibitor was found to be O-allylhydroxylamine. While O-t-butylhydroxylamine and O-benzylhydroxylamine appeared to be the best enzyme inhibitors, they were significantly less effective than O-allylhydroxylamine as growth inhibitors. Unlike other organisms examined to date [27,30], canaline was not a particularly good inhibitor of both enzyme activity and cell growth. The inhibition results for *M. tuberculosis *and *M. marinum *were very similar, with MIC results being identical or within 1 dilution. In addition, *M. tuberculosis *was found to have an MIC of 2 μg/ml for streptomycin while *M. marinum *had one of 8 μg/ml.

## Discussion

The specific aminotransferase involved in the formation of Met from KMTB has been examined in a number of eukaryotic and prokaryotic organisms [7,16,17]. However, within the low-GC content Gram-positive bacteria, only *B. subtilis*, *B. cereus*, and *B. anthracis *have been studied [16]. In all of these *Bacillus spp.*, a BCAT has been found to be responsible for catalysing the reaction, with *B. subtilis *and *B. cereus*/*B. anthracis *utilising enzymes from different aminotransferase subfamilies. No member of the high-GC content Gram-positive bacteria has been previously examined. Like *B. subtilis*, *M. tuberculosis *has been found to catalyse Met regeneration using a subfamily IIIa aminotransferase. In fact, the kinetic parameters for the two aminotransferases were almost identical. The *M. tuberculosis *BCAT had Km values of 1.77 – 2.85 mM and Vmax values of 2.58 – 4.28 μmol/min/mg protein for branched-chain amino acids and KMTB, while the *B. subtilis *YbgE had the corresponding values of 2.36 – 3.20 mM and 1.84 – 2.03 μmol/min/mg protein [16]. For branched-chain amino acids and KG, the values were 5.79 – 6.16 mM and 11.82 – 14.35 μmol/min/mg protein for the *M. tuberculosis *BCAT, and 2.82 – 3.99 mM and 13.93 – 16.61 μmol/min/mg protein for *B. subtilis *YbgE [16]. Therefore, a 45% sequence identity between the two enzymes is sufficient to conserve both the substrate range and kinetic properties of the BCATs. Structural information is only available for the *E. coli *BCAT (IlvE) and the human mitochondrial BCAT [24,24,31], but the key residues involved in substrate specificity appear to be conserved in the *M. tuberculosis *BCAT. However, while the human mitochondrial BCAT is also a family IIIa aminotransferase, there are some clear differences when compared to the *M. tuberculosis *enzyme. The human enzyme will not accept aromatic amino acids, whereas the tuberculosis BCAT would use phenylalanine as an amino donor. In addition, the human enzyme contains the redox-active motif CXXC at positions 311–314 (positions 341–344 in Figure 3) which is essential for maintaining activity, while the tuberculosis BCAT lacks these residues. Structural analysis of the *M. tuberculosis *and/or *B. subtilis *enzymes would clarify these issues.

The *M. tuberculosis *BCAT was also screened with a variety of aminooxy compounds as potential inhibitors. These compounds are known aminotransferase inhibitors and act by forming a stable Schiff-base with the PLP cofactor [32]. Unlike previous studies [7,16,17,33], canaline was not found to be one of the better inhibitors of aminotransferase activity. Instead, O-benzylhydroxylamine, O-t-butylhydroxylamine, carboxymethoxylamine, and O-allylhydroxylamine were the most efficient inhibitors of Met formation from KMTB. In addition, these compounds demonstrated mixed type inhibition with a lower Ki for the competitive component. This result contrasts with that previously found for canaline with the *Bacillus spp. *enzymes, where inhibition was uncompetitive [16]. It may be possible that this difference may be due to the structure of the inhibitors, as canaline is a γ-substituted amino acid analogue, while the present inhibitors are α-substituted or non-amino acid analogues. Essentially, the inhibitors examined in this study do not present an α-amino group suitable for participation in the transamination reaction whereas canaline does.

Further screening of the inhibitors against *M. tuberculosis *and *M. marinum *in vitro demonstrated that the compounds can act as effective antimycobacterial agents. The close correspondence of the MIC values for *M. tuberculosis *and *M. marinum *validates the use of the latter organism as a more rapid and safe initial screen of the antimycobacterial properties of aminooxy compounds. The MIC values found for streptomycin against these two organisms was also found to be consistent with previously published values [34]. *M. marinum *can thus be used to quickly test a larger number of potential inhibitors, with *M. tuberculosis *used as a follow up for more promising candidates.

Interestingly, there was no direct correlation between the Ki of the compounds against recombinant *M. tuberculosis *BCAT and the MIC/IC_50 _against cell growth. It is possible that there may be differences in the uptake rate of the various compounds into viable cells. Alternatively, the most effective growth inhibitors act by inhibiting other PLP-dependent enzymes in addition to BCAT. In any case, O-allylhydroxylamine was the most effective antimycobacterial agent with an MIC of 78 μM against *M. marinum *and 156 μM against *M. tuberculosis*. Unfortunately, the compound is corrosive, and is thus unsuitable for further in vivo study. However, the structure of the compound might provide the basis for the design of less toxic, more active structural analogues. In future studies, it will be necessary to examine the effect of potential inhibitors on human BCAT, in order to better assess the potential for host toxicity. Any further development of aminooxy compounds as antimycobacterial agents will depend on discovering a selective inhibitor for the microbial enzyme.

Several older studies have been conducted on the antimicrobial effect of aminooxy compounds, with *M. tuberculosis *included amongst the organisms tested [35-39]. From these papers, the only compound in common with the present study was carboxymethoxylamine, which was found to have an MIC of 313 μM (present data), 910 μM [35], 170 – 686 μM [36], or 170 μM [37]. Given the variety of media used in these studies for determining the MIC value, the results are quite consistent. The variety of non-commercially available aminooxy compounds synthesized and tested in these older studies included aminooxy acids, aminooxy amides, aminooxy hydroxamic acids, aminooxy hydrazides, aminooxy alkanes, and aminooxy guanidines. Several of these compounds were very effective growth inhibitors in vitro, with MIC values as low as 0.30 μM against *M. tuberculosis*. One of the compounds has been administered to mice, with favourable, albeit sparsely detailed, results with regard to toxicity and in vivo antitubercular effect [38]. While it is unclear what effect these inhibitors would have against the *M. tuberculosis *BCAT, it would appear to be possible to design more effective, less toxic aminooxy compounds for use against *M. tuberculosis*.

Several interesting findings arose during the course of this investigation. First, while *M. tuberculosis *has only the one branched-chain aminotransferase, it does contain a coding sequence (Rv0858c) with a high similarity to the *B. subtilis *ykrV gene product. YkrV was found to be a subfamily If aminotransferase and could also catalyse the conversion of KMTB to Met using glutamine as the only effective amino donor [16]. Therefore, it is possible that the Rv0858c gene product might be capable of KMTB transamination. It should be stressed that while the recombinant *B. subtilis *YkrV could transaminate KMTB with glutamine, *B. subtilis *cell homogenates did not produce Met from KMTB when supplemented with glutamine [16]. Similarly, cell homogenates of *M. smegmatis *grown in Middlebrook 7H9 incomplete medium were only able to produce Met from KMTB when supplemented with valine, isoleucine, leucine, glutamate, or phenylalanine, as was seen for the recombinant *M. tuberculosis *BCAT in figure 5 (data not shown).

*M. tuberculosis *was found to contain no putative gene product with significant homology to a DAAT. In fact, the organism appeared to contain no subfamily IIIb aminotransferases. The physiological significance of a lack of a DAAT is unclear, but many organisms do not contain a homologue of this enzyme. With DAAT, there might be a diminished capacity to catabolise D-amino acids for energy, although the same reactions could be performed by a D-amino acid oxidase. *M. tuberculosis *is known to be reliant on carbohydrate catabolism during the active growth phase and lipid metabolism during the chronic, dormant phase [40]. Therefore, the lack of a DAAT might be reflective of a lifestyle where protein and peptide catabolism is relatively unimportant.

Similarly, *M. tuberculosis *was found to lack clearly identifiable homologues of several enzymes in the Met regeneration pathway. The most glaring omission is the lack of an S-adenosylmethionine decarboxylase (SAMdc) homologue (see Figure 1). *M. tuberculosis *contains the preceding enzyme, methionine adenosyltransferase [21], and has an easily identifiable homologue for the succeeding enzyme, spermidine synthase [23]. Therefore, *M. tuberculosis *must catalyse SAMdc activity via another enzyme in order to be able to synthesize polyamines. A previous study has demonstrated SAMdc activity in *M. bovis *homogenates, but has not identified the enzyme responsible [41]. Resolution of this issue is critical for a more complete understanding of polyamine biosynthesis in tuberculosis, and may yield a novel enzyme as an additional drug target. The *M. tuberculosis *genome also appears to be missing homologues of the enzymes converting methylthioribose to KMTB (see Figure 1). However, outside of *K. pneumoniae *and *B. subtilis*, these enzymes have not been well studied, and, between these two organisms, there are key differences in the enzymes catalyzing several steps [12,20]. In silico analyses have suggested that *Pseduomonas aeruginosa*, *Xylella fastidiosa*, *Leptospira interrogans*, and *Thermoanaerobacter tengcongensis *have readily identifiable, complete Met regeneration pathways [42]. However, the presence or absence of the pathway in a variety of prokaryotic and eukaryotic organisms remains to be determined by functional analysis. Therefore, there is much left to examine before concluding that *M. tuberculosis *contains neither homologues nor analogues to these Met recycling enzymes. However, even in the absence of a complete Met salvage pathway, *M. tuberculosis*, as an intracellular pathogen, might utilise exogenous KMTB as a Met source.

## Conclusions

Branched-chain amino acid aminotransferase has been cloned and characterised from *M. tuberculosis*. This enzyme was found to be responsible for the formation of methionine from ketomethiobutyrate, and could be inhibited in vitro by a series of aminooxy compounds. Several of these compounds were found to be effective inhibitors of *M. tuberculosis *or *M. marinum *growth in culture, with MIC values as low as 156 μM and 78 μM respectively. These studies demonstrate the importance branched-chain amino acid and methionine metabolism to the survival of mycobacteria, and open up the potential for the development of more potent and less toxic aminooxy inhibitors of the branched-chain aminotransferase.

## Methods

### Cells and reagents

*M. tuberculosis *H37Rv and *M. marinum *Aronson (ATCC927) were cultured in liquid Middlebrook 7H9 complete medium or on Middlebrook 7H10 plates at 37°C for *M. tuberculosis *or 30°C for *M. marinum*. All substrates and inhibitors were obtained from Sigma-Aldrich (Oakville, ON, Canada).

### Cloning and functional expression

Genomic DNA was isolated from *M. tuberculosis *by vortexing packed cells in a minimal volume of 50 mM Tris-HCl pH 8.0/10 mM EDTA/100 mM NaCl containing 500 μm acid washed glass beads (Sigma). After allowing the glass beads to settle, the supernatant was added to an equal volume of 10 mM Tris-HCl pH 8.0/100 mM NaCl/25 mM EDTA/0.5% w/v sodium dodecyl sulfate/0.1 mg/ml proteinase K and incubated for 1 hr at 37°C with occasional gentle mixing. The mixture was then subjected to extraction with phenol:chloroform:isoamyl alcohol (25:24:1), and the DNA ethanol precipitated.

The sequence of the putative *M. tuberculosis *BCAT gene was discovered by a BLAST search of the complete *M. tuberculosis *H37Rv genome using the *B. subtilis *YbgE, YwaA, or YheM gene products as the query proteins [16,23,43]. The single resulting putative BCAT gene was used to construct oligonucleotide primers for PCR amplification. The 5' primer was TCGAGGCGGCCGCAAATGACCAGCGGCTCCCTTCA and incorporated a NotI restriction site and an in-frame start codon. The 3' primer was ATCGAGCTCGAGTTACCCCAGCCGCGCCATCCAG and incorporated a XhoI restriction site and an in-frame stop codon. The BCAT gene was then amplified using a 5:1 mixture of Taq:Pfu polymerases (Promega; Madison, WI, USA) and the following program: 1 cycle of 95°C for 1.5 min; 30 cycles of 95°C for 1 min, 55°C for 1 min, and 72°C for 1 min; and 1 cycle of 72°C for 10 min. The resulting PCR product was excised from a 1% agarose gel and recovered using the Qiaex II kit (Qiagen; Mississauga, ON, Canada). The purified product was digested with NotI and XhoI and ligated into a similarly digested pET 19 m (a modification by us of pET19b (Novagen; Madison, WI, USA) to incorporate extra restriction sites in the multiple cloning site) using a Rapid Ligation kit (Fermentas; Burlington, ON, Canada). The recombinant plasmid was then transformed into *Escherichia coli *XL10 cells (Stratagene; La Jolla, CA, USA) and was subsequently recovered using the Qiaspin miniprep kit (Qiagen). Positive clones were determined by digesting the plasmid with NotI and XhoI to confirm the presence of the insert on a 1% agarose gel. The sequence of the insert was confirmed by using the Big-Dye cycle sequencing kit (ABI; Foster City, CA, USA) and an ABI Prism 310 genetic analyser.

The plasmid from positive clones was transformed into *E. coli *BL21(DE3) CodonPlus RIL cells (Stratagene) for functional expression. Cells were grown in liquid LB medium containing 50 μg/ml ampicillin and 50 μg/ml chloramphenicol at 37°C and 250 rpm until the culture reached an A_600 nm _of 0.6–0.8. The culture was then cooled to 20°C for 30 min at 250 rpm before the addition of 0.1 mM isopropylthiogalactopyranoside (IPTG) and an additional 20 hr of incubation at 20°C and 250 rpm.

The culture was centrifuged at 3500 × g for 20 min at 4°C, and the cell pellet resuspended in 50 mM HEPES (pH 7.4)/750 mM NaCl and frozen at -20°C. The resuspended cells were then thawed, sonicated on ice, centrifuged at 3000 × g for 20 min at 4°C, and the supernatant loaded onto a 1.6 × 9.5 Chelating-Sepharose-FF column (Amersham Biosciences; Baie d'Urfe, QC, Canada) charged with NiSO_4_. The column was washed with 50 mM HEPES (pH 7.4)/750 mM NaCl and 50 mM HEPES (pH 7.4)/750 mM NaCl/80 mM imidazole, before elution with 50 mM HEPES (pH 7.4)/750 mM NaCl/800 mM imidazole. Fractions containing the recombinant protein were pooled and concentrated to less than 3.0 ml using a 30 kDa molecular mass cut-off filter (Pall Filtron; Mississauga, ON, Canada). The concentrated enzyme was then dialysed against 50 mM HEPES (pH 7.4)/1 mM dithiothreitol/1 mM EDTA/trace pyridoxal-5-phosphate (PLP) overnight at 4°C. The concentrated enzymes were stored at 4°C for several days, or with 20% v/v glycerol at -20°C for several weeks, without appreciable loss of activity. Recombinant protein samples were examined by electrophoresis on 10% SDS polyacrylamide gels followed by Coomassie Brilliant Blue R250 staining. Protein concentration was measured using the Bio-Rad reagent (Bio-Rad; Mississauga, ON, Canada).

### Enzyme assays and inhibition studies

Aminotransferase activities were assayed by an HPLC method [17]. 5 or 10 μl of recombinant enzyme was added to 100 μl of substrate mix (100 mM PO_4 _(pH 7.4)/50 μM PLP/various concentrations of amino acid/various concentrations of keto acid) and incubated for 30 min at 37°C. The samples were then stored at -20°C until analysis by HPLC. All samples were analysed by pre-column derivatisation and reverse-phase HPLC. 10 μl of sample was mixed with 50 μl of 400 mM borate pH 10.5 and then with 10 μl of 10 mg/ml o-phthalaldehyde/12 μl/ml mercaptopropionate/400 mM borate pH 10.5 prior to the injection of 7.0 μl onto a 2.1 × 200 mm ODS-AA column (Agilent; Mississauga, ON, Canada). The column was eluted using 2.72 mg/ml sodium acetate pH 7.2/0.018% v/v triethylamine/0.3% v/v tetrahydrofuran as Buffer A and 2.72 mg/ml sodium acetate pH 7.2/40% v/v methanol/40% v/v acetonitrile as Buffer B with a linear gradient of 0 – 17% B over 16 min followed by a linear gradient of 17–100% B over 1 min and 6.0 min at 100% B. The flow rate was 0.45 ml/min from 0 – 16 min and 0.80 ml/min from 17–30 min. The elution of derivatised amino acids was monitored at 338 nm and fluorometrically with an excitation of 338 nm and an emission of 450 nm. All separations were performed on an Agilent 1100 HPLC equipped with an autosampler, variable wavelength ultraviolet/visible spectrophotometric detector, fluorescence detector, and Chemstation operating system.

The amino donor range for Met regeneration was determined by incubating 2 mM of each individual amino acid and 1 mM KMTB, followed by HPLC for Met quantification. Amino acids which were effective amino donors were further studied at 0.1 – 10 mM amino acid and 10 mM KMTB to determine the kinetic constants. Similar assays were performed with 0.1 – 10 mM KMTB and 10 mM Leu. Replacement of KMTB with ketoglutarate (KG) in these experiments and subsequent HPLC analysis of Glu formation allowed for the determination of BCAT activity. The apparent Km and Vmax values for each substrate were assessed by non-linear curve fitting using the Scientist software programmed with the Michaelis-Menton equation (Micromath; Salt Lake City, UT, USA).

Initial inhibition studies screened 13 aminooxy compounds against *M. tuberculosis *BCAT using 2.0 mM Leu/1.0 mM KMTB/0.1 or 1.0 mM inhibitor in the enzyme incubation. Inhibitors which demonstrated better than 50% reduction of activity at the 0.1 mM concentration were further studied for the determination of K_i _values. These reactions involved 0.5, 1.0, 2.0, or 3.0 mM Leu and 1.0 mM KMTB in the reaction mixture together with 0, 25, 50, 75, 100, 150, 200 μM of inhibitor. The K_i _values were determined by non-linear curve fitting with the Scientist software programmed with competitive, uncompetitive, and mixed inhibition equations [25].

In vitro growth inhibition studies were performed on *M. tuberculosis *and *M. marinum *using the most effective enzyme inhibitors. Cultures at mid-log growth in Middlebrook 7H9 complete medium was diluted to 2 × 10^5 ^cfu/ml and 100 μl added to 96 well microtitre plates containing 100 μl of doubling dilutions of each inhibitor. The final inhibitor concentration ranged from 10 mM – 298 pM. Positive and negative controls consisted of 100 μl Middlebrook 7H9 medium replacing the inhibitor or cells respectively. The plates were incubated at 30°C for 8 days (*M. marinum*) or 37°C for 14 days (*M. tuberculosis*) with no agitation before measurement of growth at A_650 nm _using a Molecular Devices 96-well spectrophotometer (Sunnyvale, CA, USA). The MIC was determined as the lowest dilution that completely prevented microbial growth and the IC_50 _was determined by non-linear curve fitting with the Scientist software programmed with the Chou equation [44].

### Phylogenetic analysis

Additional BCAT and DAAT sequences were obtained from GenBank [38]. *Mycobacterium spp. *BCAT sequences from preliminary genome projects were made available from The Institute for Genomic Research  for *M. smegmatis *and *M. avium*, from The Sanger Centre  for *M. marinum*, and from The Institut Pasteur  for *M. ulcerans*. These sequences were aligned using the Clustal algorithm and the BLOSUM sequence substitution table in the ClustalX program [46]. Aligned sequences were viewed using the Bioedit program [47] and were then used with the ProtDist component of the PHYLIP [48] to construct a distance matrix that was the basis for tree construction using the neighbour-joining method [49]. All trees were visualised using Treeview [50].

## Authors' contributions

ESV performed the cloning, expression, and characterisation of the enzyme, and assisted in writing the manuscript. CLR assisted in the cloning and expression experiments. MHK performed the *M. marinum *experiments. BJB conceived the study, performed the *M. tuberculosis *experiments, and wrote the manuscript.

## Figures and Tables

**Table 1:** Kinetic characterization of *M. tuberculosis *branched-chain aminotransferase. The enzyme was incubated with varying concentrations of substrate and 10 mM cosubstrate, as described in the Methods section.

Substrate	Cosubstrate	Apparent Km (mM)	Apparent Vmax (μmol/min/mg protein)
Leu	KMTB	2.50 ± 0.90	3.65 ± 0.43
Val	KMTB	1.77 ± 0.86	2.58 ± 0.41
Ile	KMTB	2.85 ± 0.56	4.28 ± 0.32
Glu	KMTB	9.53 ± 3.43	5.70 ± 1.20
Phe	KMTB	7.44 ± 1.40	2.17 ± 0.22
KMTB	Leu	4.20 ± 1.79	4.22 ± 0.72
Leu	KG	6.02 ± 0.94	13.44 ± 0.84
Val	KG	5.79 ± 0.99	11.82 ± 0.80
Ile	KG	6.16 ± 1.14	14.35 ± 1.08
KG	Leu	6.95 ± 1.44	12.80 ± 1.12

**Table 2:** Ki determination for selected aminooxy inhibitors. The enzyme was incubated with variable amounts of leucine and inhibitor and fixed amounts of KMTB, as described in the Methods section. Kic and Kiu refer to the competitive and uncompetitive components of mixed-type inhibition [25].

Inhibitor	Kic(μM)	Kiu(μM)
*O*-(t-butyl)hydroxylamine	11.02 ± 2.76	85.60 ± 44.79
carboxymethoxylamine	20.97 ± 7.27	142.42 ± 69.76
*O*-allylhydroxylamine	21.61 ± 11.08	>200 (386)*
*O*-benzylhydroxylamine	8.20 ± 2.56	84.08 ± 31.91

*200 μM was the highest concentration of inhibitor tested in these experiments. The calculated Kiu value is shown in parentheses.

**Table 3:** In vitro growth inhibition of *M tuberculosis *and *M. marinum *by aminooxy compounds. One hundred μL of a mid-logarithmic culture of *M. tuberculosis *or *M. marinum *at a concentration of 2 × 10^5 ^cfu/ml was added to 100 μL of serial doubling dilutions of inhibitor in a 96-well microtitre plate. The drug plates were grown for 14 days at 37°C (*M. tuberculosis*) or 8 days at 30°C (*M. marinum*) with no agitation before checking for cell growth at A_650 nm_. The minimum inhibitory concentration (MIC) and inhibitory concentration 50% (IC50) were calculated as described in the Methods section.

Inhibitor	*M. marinum *(n = 8)		*M. tuberculosis *(n = 6)	
	
	MIC (mM)	IC50 (μM)	MIC (mM)	IC50 (μM)
*O*-allylhydroxylamine	0.078	8.49 ± 1.96	0.156	39.22 ± 2.01
carboxymethoxylamine	0.313	89.32 ± 7.65	0.313	70.99 ± 7.83
*O*-benzylhydroxylamine	1.25	410.76 ± 67.10	1.25	467.54 ± 62.80
canaline	1.25	335.29 ± 13.64	1.25	273.18 ± 14.64
*O*-(t-butyl)hydroxylamine	10	43.18 ± 10.51	10	130.52 ± 23.10
